# Neurochemical and Neuroanatomical Identification of Central Pattern Generator Neuron Homologues in Nudipleura Molluscs

**DOI:** 10.1371/journal.pone.0031737

**Published:** 2012-02-20

**Authors:** Joshua L. Lillvis, Charuni A. Gunaratne, Paul S. Katz

**Affiliations:** Neuroscience Institute, Georgia State University, Atlanta, Georgia, United States of America; Freie Universitaet Berlin, Germany

## Abstract

Certain invertebrate neurons can be identified by their behavioral functions. However, evolutionary divergence can cause some species to not display particular behaviors, thereby making it impossible to use physiological characteristics related to those behaviors for identifying homologous neurons across species. Therefore, to understand the neural basis of species-specific behavior, it is necessary to identify homologues using characteristics that are independent of physiology. In the Nudipleura mollusc *Tritonia diomedea*, Cerebral Neuron 2 (C2) was first described as being a member of the swim central pattern generator (CPG). Here we demonstrate that neurochemical markers, in conjunction with previously known neuroanatomical characteristics, allow C2 to be uniquely identified without the aid of electrophysiological measures. Specifically, C2 had three characteristics that, taken together, identified the neuron: 1) a white cell on the dorsal surface of the cerebral ganglion, 2) an axon that projected to the contralateral pedal ganglion and through the pedal commissure, and 3) immunoreactivity for the peptides FMRFamide and Small Cardioactive Peptide B. These same anatomical and neurochemical characteristics also uniquely identified the C2 homologue in *Pleurobranchaea californica* (called A1), which was previously identified by its analogous role in the *Pleurobranchaea* swim CPG. Furthermore, these characteristics were used to identify C2 homologues in *Melibe leonina*, *Hermissenda crassicornis*, and *Flabellina iodinea*, species that are phylogenetically closer to *Tritonia* than *Pleurobranchaea*, but do not display the same swimming behavior as *Tritonia* or *Pleurobranchaea*. These identifications will allow future studies comparing and contrasting the physiological properties of C2 across species that can and cannot produce the type of swimming behavior exhibited by *Tritonia*.

## Introduction

**Figure 1:**
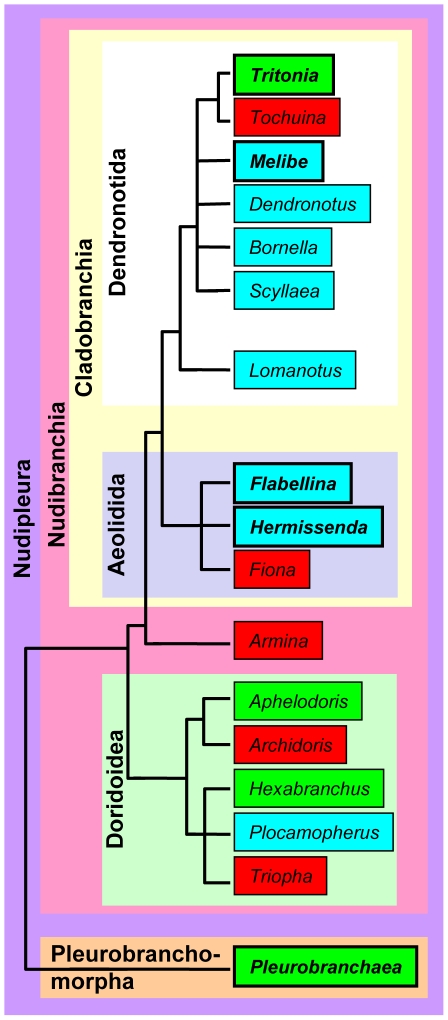
An abbreviated phylogeny of the Nudipleura Clade within Opisthobranchia. The Nudipleura clade consists of the monophyletic clades Nudibranchia and Pleurobranchomorpha. Within Nudibranchia is the clade Cladobranchia, which consists of Dendronotida and Aeolida. Nudibranchia also includes the clade Doridoidea. The genera studied here are in bold. Genera in green boxes are genera that include species that produce rhythmic, dorsal-ventral flexions, in turquoise boxes are genera that produce rhythmic, left-right flexions, and in red boxes have not been observed to produce rhythmic body flexions. Larger boxes indicate monophyletic clades.

The ability to reliably identify neurons in some invertebrate model systems allows individual neurons to be linked to behavior. As such, it is compelling to attempt to find homologues of identified neurons across species with similar or different suites of behavior. This allows the physiological properties of the homologues to be compared in an effort to better understand the neural basis of behavior and its evolution. It is not possible to use behavioral function to characterize neuronal homologues, however, if the behavior used to identify the neuron differs across species. Therefore, characteristics must be found that can identify homologous neurons regardless of physiological activity. Here, as a step toward comparing the physiological properties of homologous neurons in species with different behaviors, we have used neuroanatomical and neurochemical characteristics to identify homologues of an individual neuron across gastropod mollusc species within the Nudipleura clade (Figure 1) [1].

Gastropod molluscs are well suited for a comparative study of single neurons and small neural networks. The phylogeny is well documented [1]–[6] and closely related species display a variety of behaviors [7]–[9]. Moreover, gastropod brains have a relatively small number of neurons (5,000–10,000), which have large cell bodies (up to 1 mm in diameter) [10], [11]. Many gastropod neurons can be uniquely identified based upon a suite of anatomical, neurochemical, and electrophysiological characteristics [12], [13]. Homologues of neurons in other species can be identified using the same suite of characteristics [12], [14]–[24]. The argument for homology is based on parsimony; it being more likely that neurons with the same identifying characteristics in different species were present in a common ancestor than that they acquired these uniquely identifying characteristics independently [12], [25]. The more species that exhibit the shared characteristics, the stronger is the argument in favor of homology. The ability to identify homologues in gastropods allows comparative studies of homologous neurons across species with similar or divergent suites of behavior. Studies of this kind have been conducted to comparatively investigate the neural basis of feeding [14]–[18], [22], [26], [27] and locomotor [19], [20], [23], [28] behaviors across species.

The species to which others will be compared is a gastropod mollusc within the Nudipleura clade named *Tritonia diomedea*. *Tritonia* produces a swim that consists of rhythmic, alternating dorsal and ventral whole body flexions [29]–[31]. The central pattern generator (CPG) underlying the swim is made up of just three cell types: the Dorsal Swim Interneurons (DSI) (http://neuronbank.org/Tri0001043), Ventral Swim Interneurons (VSI) (http://neuronbank.org/Tri0002436), and Cerebral Neuron 2 (C2) (http://neuronbank.org/Tri0002380) [32]–[36]. In an effort to understand how *Tritonia* can produce a dorsal-ventral swim while most Nudipleura molluscs cannot, we wanted to identify homologues of CPG neurons across species that can and cannot swim like *Tritonia* (Figure 1). This will enable future studies comparing the properties of the homologous neurons. Previous work identified homologues of DSI in 11 species based on conserved soma location, axon projection, and serotonergic immunoreactivity [20], [21], [27], [37]–[41]. Here, we sought to identify another CPG neuron, C2, across species.

We have established neurochemical characteristics that allow C2 to be uniquely identified in *Tritonia* without the aid of electrophysiological measures. Using these characteristics, we have provided further evidence for homology of the previously identified A1 neuron (http://neuronbank.org/Ple0002601) in *Pleurobranchaea californica*, a species that can swim like *Tritonia* [19], [20]. Furthermore, we found that the same characteristics uniquely identified C2 homologues in three Nudipleura molluscs that cannot swim like *Tritonia*: *Melibe leonina*, *Hermissenda crassicornis*, and *Flabellina iodinea*. Similar results were reported by Longley and Longley in a 1987 abstract for the Society for Neuroscience annual meeting.

## Results

### Identifying C2 in Tritonia diomedea

**Figure 2:**
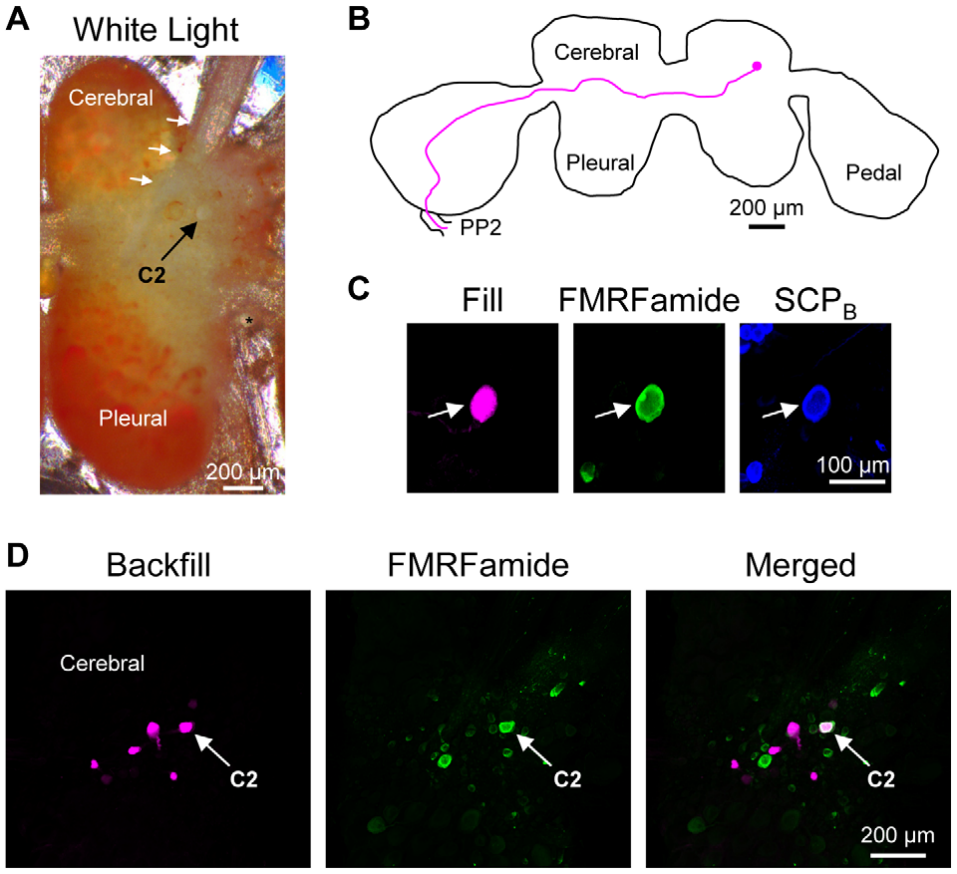
C2 characteristics in *Tritonia diomedea*. **A.** C2*_Tri_* could be identified visually due to its characteristic white soma near the origin of cerebral nerve 1 (CeN1, white arrows). An asterisk (*) labels the statocyst. Only one cerebral-pleural ganglion is shown. **B.** Filling the C2*_Tri_* soma with Neurobiotin revealed a contralateral axon projection through the anterior cerebral-pedal commissure (not shown) and into the pedal commissure (PP2). The example is a representative image in which the outline of the brain and the axon projection were traced for ease of viewing. **C.** C2*_Tri_* (arrow) was filled with biocytin (left). It was immunoreactive for both FMRFamide (middle) and SCP_B_ (right) as shown. **D.** Backfilling the pedal commissure with biocytin in *Tritonia* labeled 3–4 neurons near C2*_Tri_* (left). Only the cerebral-pleural ganglion contralateral to the backfilled nerve is shown. FMRFamide-like immunohistochemistry labeled C2*_Tri_* (middle). Combining FMRFamide-like immunoreactivity with the backfill revealed just one neuron, C2*_Tri_*, (right).

The *Tritonia* brain consists of paired fused cerebral, pleural and pedal ganglia (Figure 2A, B) [42]. C2*_Tri_* (subscripts will be used here to distinguish homologues in each species) is a bilaterally represented neuron with a white soma found on the dorsal surface of the cerebral ganglion. The soma is located in the anterior-lateral region of the ganglion near the origin of cerebral nerve 1 (CeN1), a nerve that originates on the dorsal surface of the cerebral ganglion [42] (Figure 2A) (nerve nomenclature is based on reference [43] unless indicated otherwise). C2*_Tri_* has a characteristic contralateral axon projection to the pedal ganglion and through the pedal commissure (PP2; pedal nerve 6), the largest of the commissure nerves that connect the left and right pedal ganglia (Figure 2B, D) [32], [44]. These anatomical features help to identify C2*_Tri_*, but electrophysiological characters are often necessary to unequivocally identify the neuron. Specifically, C2 receives spontaneous, discrete excitatory post-synaptic potentials but is quiescent at rest. It is also electrically coupled to its contralateral counterpart [33]. Finally, synaptic connections with other swim CPG members and the participation of C2 in the dorsal-ventral swim CPG unequivocally identify this neuron [35], [45], [46]. Here, we have further characterized C2*_Tri_* using anatomical and neurochemical measures in order to allow homologue identification in species that may not share the electrophysiological properties observed in **Tritonia**.

Neurochemical markers can greatly aid in identifying individual neurons both within and across species. Evidence suggests that C2*_Tri_* is peptidergic [47] and our immunohistochemistry experiments support that hypothesis. We filled the C2*_Tri_* soma with a biotinylated tracer and tested antisera and monoclonal antibodies raised against neuropeptides on whole brain preparations. We determined that C2*_Tri_* displays dual FMRFamide-like immunoreactivity (n = 10) and Small Cardioactive Peptide B (SCP_B_)-like immunoreactivity (n = 6 somata in 5 preparations) (Figure 2C).

C2*_Tri_* could be unequivocally identified with just FMRFamide immunoreactivity in conjunction with the contralateral axon projection to PP2. Backfilling PP2 with biocytin showed that approximately four cell bodies in the vicinity of C2*_Tri_* share a similar contralateral axon projection to PP2 (Figure 2D; n = 4). However of the four cell bodies, only C2*_Tri_* also displayed FMRFamide immunoreactivity (Figure 2D, n = 3). Moreover, C2*_Tri_* was the only neuron on the dorsal surface of the cerebral-pleural ganglion that was both FMRFamide immunoreactive and projected an axon contralaterally to and through PP2. Thus, C2*_Tri_* can be uniquely identified without the aid of electrophysiological characters; the characteristics of a FMRFamide immunoreactive soma on the dorsal surface of the cerebral-pleural ganglion in conjunction with a contralateral axon projection into PP2 were sufficient to identify the neuron. Additional characteristics of C2 were SCP_B_ immunoreactivity and a contralateral axon projection through the anterior of two fiber tracts connecting the cerebral and pedal ganglia (anterior cerebral-pedal commissure) before reaching PP2. Furthermore, the characteristics of a white soma on the dorsal surface of the brain near the origin of CeN1 help to identify the neuron in the living preparation.

### Identifying C2 in Pleurobranchaea californica

**Figure 3:**
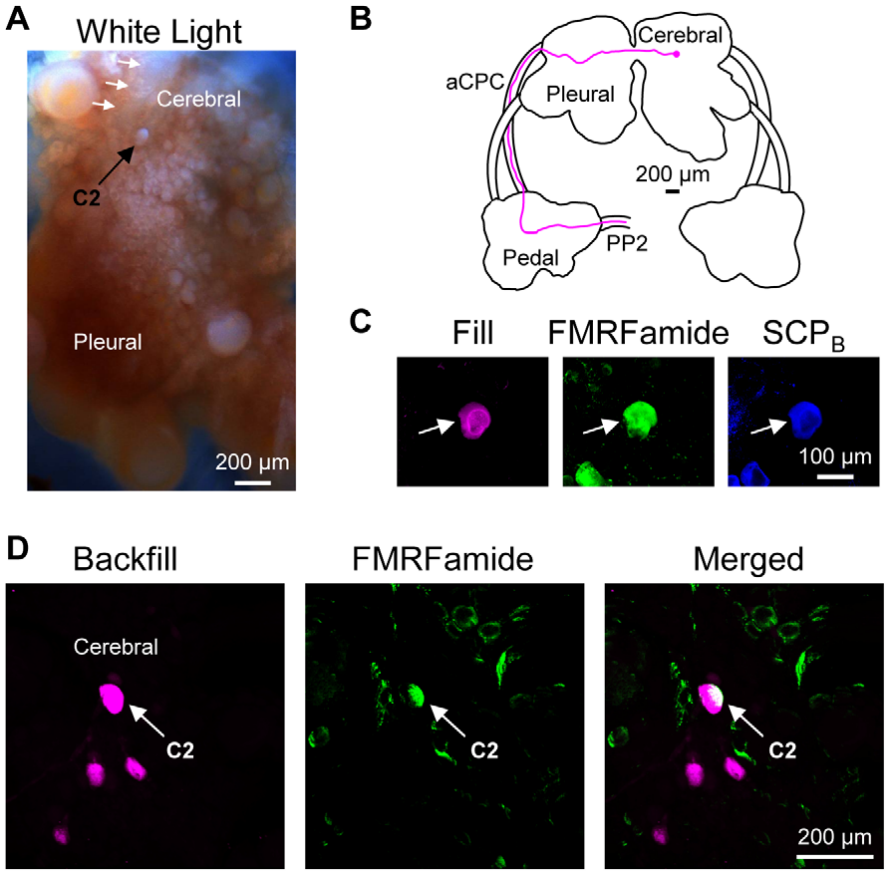
C2 characteristics in *Pleurobranchaea californica*. **A.** C2*_Pleur_* could be identified visually due to its characteristic white soma near the origin of cerebral nerve 1 (CeN1, white arrows). It was the more anterior-lateral of two white neurons near the origin of CeN1 (the more medial white neuron is not seen here). Only one cerebral-pleural ganglion is shown. **B.** Filling the C2*_Pleur_* soma with biocytin revealed a contralateral axon projection through the anterior cerebral-pedal commissure (aCPC) into the pedal commissure (PP2). The example is a representative image in which the outline of the brain and the axon projection were traced for ease of viewing. **C.** C2*_Pleur_* (arrow) was filled with biocytin (left). It was immunoreactive for both FMRFamide (middle) and SCP_B_ (right) as shown. **D.** Backfilling the pedal commissure with biocytin labeled 3–4 neurons near C2*_Pleur_* (left). Only the cerebral-pleural ganglion contralateral to the backfilled nerve is shown. FMRFamide-like immunohistochemistry labeled C2*_Pleur_* (middle). Combining FMRFamide-like immunoreactivity with the backfill revealed just one neuron, C2*_Pleur_* (right).

*Pleurobranchaea* is in the Pleurobranchomorpha clade of Nudipleura, which makes it the species that is most distantly related to *Tritonia* in this study (Figure 1) [1]. It is the only other species investigated, however, that can produce a rhythmic, dorsal-ventral swim like that of *Tritonia* [48] (Figure 1). The CPG underlying the *Pleurobranchaea* swim contains homologues of the *Tritonia* swim CPG neurons, including the homologue of C2 [19], [20], which was named A1, but will be called C2*_Pleur_* here for consistency. C2*_Pleur_* is not only part of the swim CPG, but also has a white soma near the origin of CeN1 (called the rhinophore nerve in *Pleurobranchaea*) (Figure 3A) and a contralateral axon projection through the anterior cerebral-pedal commissure and through PP2 (called the pedal commissure in *Pleurobranchaea*) (Figure 3B, D) [19].

Experiments in which the C2*_Pleur_* soma was injected with a biotinylated tracer combined with whole brain immunohistochemistry revealed that, like C2*_Tri_*, C2*_Pleur_* displayed dual FMRFamide (n = 11 somata in 7 preparations) and SCP_B_ immunoreactivity (n = 7 somata in 4 preparations) (Figure 3C). Moreover, combining biocytin backfills of PP2 with FMRFamide immunohistochemistry, showed that C2*_Pleur_* was the only neuron on the dorsal surface of the cerebral-pleural ganglion with a contralateral axon projection to and through PP2 that is also immunoreactive for FMRFamide (n = 6) (Figure 3D). Consequently, the same anatomical and neurochemical characteristics that could uniquely identify C2*_Tri_* also uniquely identified C2 in *Pleurobranchaea*, the most distantly related species investigated here.

### Identifying C2 in Melibe leonina

Like *Tritonia*, *Melibe leonina* is a dendronotid within the nudibranch clade of Nudipleura, making *Melibe* the species that is mostly closely related to *Tritonia* in this study (Figure 1) [1]. *Melibe* can perform a swim consisting of rhythmic, left-right whole- body flexions [49], but not a dorsal-ventral swim like that of *Tritonia* or *Pleurobranchaea*.

**Figure 4:**
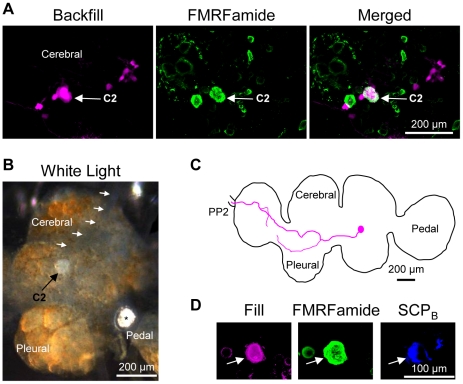
C2 characteristics in *Melibe leonina*. **A.** Backfilling the pedal commissure with biocytin labeled 3–4 neurons near C2*_Mel_* (left). Only the cerebral-pleural ganglion contralateral to the backfilled nerve is shown. FMRFamide-like immunohistochemistry labeled C2*_Mel_* (middle). Combining FMRFamide-like immunoreactivity with the backfill revealed just one neuron, C2*_Mel_* (right). **B.** C2*_Mel_* could be identified visually due to its characteristic white soma near the origin of cerebral nerve 1 (CeN1, white arrows). An asterisk (*) labels the statocyst. Only one cerebral-pleural ganglion is shown. **C.** Filling the C2*_Mel_* soma with Neurobiotin revealed a branching contralateral axon projection through the anterior cerebral-pedal commissure (not shown) and into the pedal commissure (PP2). The example is a representative image in which the outline of the brain and the axon projection were traced for ease of viewing. **D.** C2*_Mel_* (arrow) was filled with biocytin (left). It was immunoreactive for both FMRFamide (middle) and SCP_B_ (right) as shown.

Biocytin backfills of PP2 in conjunction with FMRFamide immunohistochemistry uniquely identified a neuron in *Melibe* with the same characteristics as C2*_Tri_*, which we have named C2*_Mel_* (Figure 4A). As in *Tritonia* and *Pleurobranchaea*, C2*_Mel_* was the only neuron on the dorsal surface of the cerebral-pleural ganglion with a contralateral axon projection into PP2 that was also FMRFamide immunoreactive (n = 5). The soma of C2*_Mel_* was also white, but relatively more posterior-medial than the position of the C2 soma in *Tritonia* and *Pleurobranchaea* (Figure 4B). However, the origin of CeN1 is more posterior-medial in *Melibe* as well. Thus, the C2*_Mel_* soma position in relation to the origin of CeN1 was similar to that of the other species tested.

Injecting a biotinylated tracer into the soma of C2*_Mel_* combined with FMRFamide and SCP_B_ immunohistochemistry confirmed that the neuron was FMRFamide (n = 7) and SCP_B_ (n = 3) immunoreactive (Figure 4D). Tracer injection also revealed a more detailed axon projection. While the axon projection was similar to C2 in the other species tested, in that it has a contralateral projection through the anterior cerebral-pedal commissure and then into PP2, there were additional branches not observed in the other species (n = 4) (Figure 4C). Still, the characteristics of neurochemical staining and axon projection that identified C2 in the other species also uniquely identified C2*_Mel_*; no other neuron exhibited these features. We therefore conclude that C2*_Mel_* is homologous to C2*_Tri_*.

### Identifying the C2 homologue in Hermissenda crassicornis

*Hermissenda* is an aeolid within the nudibranch clade and Cladobranchia sub-clade (Figure 1) [1]. As such, it is more closely related to *Tritonia* and *Melibe* than it is to *Pleurobranchaea*. We observed that *Hermissenda* was able to produce rhythmic, left-right whole-body flexions, however the behavior was not as robust as the swim observed in *Melibe* in that it did not cause the animal to stay suspended in the water. The response was not observed in all of the individual *Hermissenda* tested; in one trial, 6 of 11 animals produced these body flexions in response to a 100 µl puff of 5 M NaCl.

**Figure 5:**
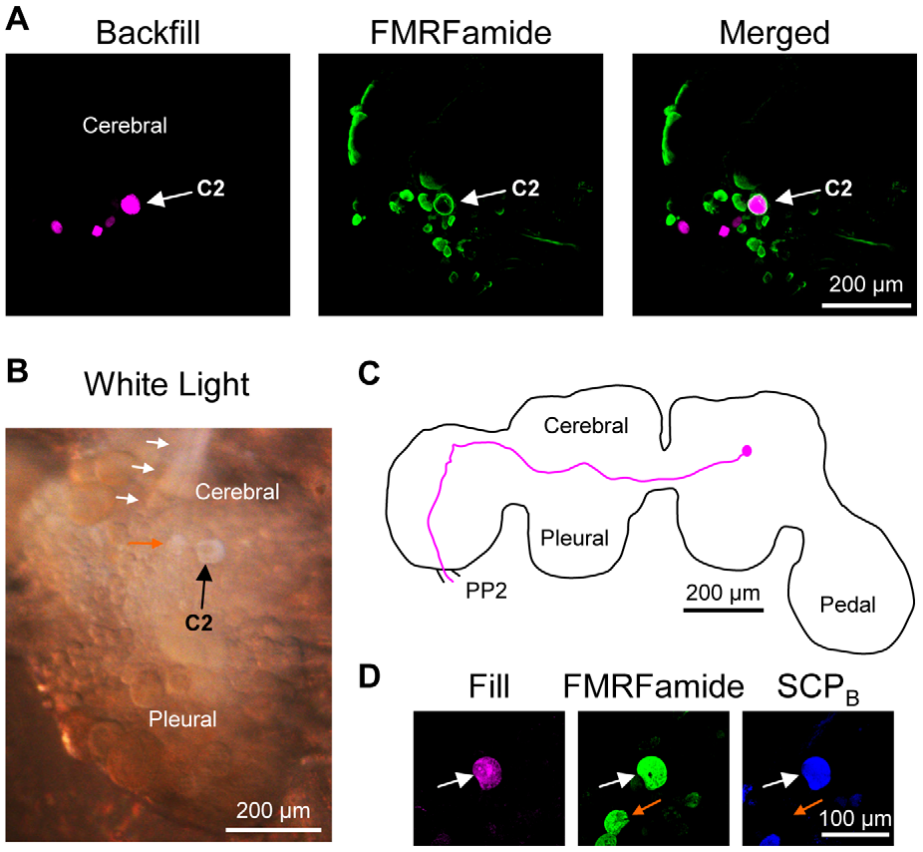
C2 characteristics in *Hermissenda crassicornis*. **A.** Backfilling the pedal commissure with biocytin labeled 3–4 neurons near C2*_Herm_* (left). Only the cerebral-pleural ganglion contralateral to the backfilled nerve is shown. FMRFamide-like immunohistochemistry labeled C2*_Herm_* (middle). Combining FMRFamide-like immunoreactivity with the backfill revealed just one neuron, C2*_Herm_* (right). **B.** C2*_Herm_* could be identified visually due to its characteristic white soma (black arrow) near the origin of cerebral nerve 1 (CeN1, white arrows). Note the white cell just medial to C2*_Herm_* (orange arrow). An asterisk (*) labels the statocyst. Only one cerebral-pleural ganglion is shown. **C.** Filling the C2*_Herm_* soma with biocytin revealed a contralateral axon projection through the anterior cerebral-pedal commissure (not shown) and into the pedal commissure (PP2). The example is a representative image in which the outline of the brain and the axon projection were traced for ease of viewing. **D.** C2*_Herm_* (arrow) was filled with biocytin (left). It was immunoreactive for both FMRFamide (middle) and SCP_B_ (right) as shown. The white cell just medial to C2*_Herm_* is immunoreactive for FMRFamide, but not SCP_B_ (orange arrow).

As in the other species investigated, biocytin backfills of PP2 in conjunction with FMRFamide immunohistochemistry revealed that only one neuron on the dorsal surface of the *Hermissenda* cerebral-pleural ganglion had a contralateral axon projection into PP2 and displayed FMRFamide immunoreactivity (n = 3) (Figure 5A). Filling the white soma of this neuron (Figure 5B), which we will now call C2*_Herm_*, showed that it displayed dual FMRFamide (n = 11) and SCP_B_ immunoreactivity (n = 7 somata in 5 preparations) (Figure 5D), and a contralateral axon projection through the anterior cerebral-pedal commissure and into PP2 (Figure 5C). We conclude from this evidence that C2*_Herm_* is homologous to C2*_Tri_*. It should be noted, that there was another neuron with a white soma near the origin of CeN1 that was FMRFamide immunoreactive (Figure 5D) and had a contralateral axon projection to the pedal ganglion. The axon did not project into PP2 and the neuron was not SCP_B_ immunoreactive, however (Figure 5D). An additional characteristic to help identify C2*_Herm_* from the nearby white soma in the living preparation is that C2*_Herm_* is the more anterior-lateral of the two white cells near CeN1 (Figure 5B, D).

### Identifying C2 in Flabellina iodinea

*Flabellina* is an aeolid, like *Hermissenda*, and thus shares the same phylogenetic relationship to *Tritonia* (Figure 1) [1]. *Flabellina* demonstrates a much more robust left-right, rhythmic swimming behavior than *Hermissenda*, however [7].

**Figure 6:**
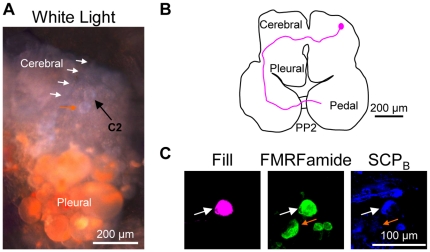
C2 characteristics in *Flabellina iodinea*. **A.** C2*_Flab_* could be identified visually due to its characteristic white soma (black arrow) near the origin of cerebral nerve 1 (CeN1, white arrows). Note the white cell just posterior-medial to C2*_Flab_* (orange arrow). Only one cerebral-pleural ganglion is shown. **B.** Filling the C2*_Flab_* soma with biocytin revealed a contralateral axon projection through the anterior cerebral-pedal commissure (not shown) and into the pedal commissure (PP2). The example is a representative image in which the outline of the brain and the axon projection were traced for ease of viewing. **C.** C2*_Flab_* (arrow) was filled with biocytin (left). It was immunoreactive for both FMRFamide (middle) and SCP_B_ (right) as shown. The white cell just medial to C2*_Herm_* is immunoreactive for FMRFamide, but not SCP_B_ (orange arrow).

As in *Hermissenda*, there were two white neurons near the origin of CeN1 that displayed FMRFamide immunoreactivity (Figure 6A). Biotinylated tracer fills of both of these white cells revealed that only one, which we now call C2*_Flab_*, demonstrated dual immunoreactivity for FMRFamide and SCP_B_ and also had a contralateral axon projection through the anterior cerebral-pedal commissure and into PP2 (n = 4) (Figure 6B, C). As in *Hermissenda*, this was the more anterior-lateral of the two white cells (Figure 6A, C). Also like *Hermissenda*, the more medial white cell had a contralateral axon projection to the pedal ganglion and was FMRFamide immunoreactive but did not display SCP_B_ immunoreactivity (Figure 6C) or an axon projection into PP2. Unfortunately, PP2 in *Flabellina* was too short to effectively backfill with biocytin. However, the suite of characteristics that uniquely identify C2 in *Tritonia*, *Pleurobranchaea*, *Melibe*, and *Hermissenda* also identified C2*_Flab_*. Therefore, we conclude that C2*_Flab_* is homologous to C2*_Tri_*.

## Discussion

Neurochemical and neuroanatomical characteristics were found to uniquely identify C2 in *Tritonia* and homologues in four other Nudipleura molluscs independent of electrophysiological activity. C2*_Tri_* can be uniquely identified as a neuron having the following characteristics: 1) a white cell body near the origin of CeN1 on the dorsal surface of the cerebral ganglion, 2) FMRFamide and SCP_B_ immunoreactivity, and 3) an axon that projects contralaterally through the anterior cerebral-pedal commissure to the pedal ganglion and then through PP2. We showed that these same three characteristics can be used to identify the previously described C2 homologue in *Pleurobranchaea californica* [19]. Homology is further supported by the fact that C2*_Tri_* and C2*_Pleur_* share electrophysiological characteristics, such as activity during a swim motor pattern and synaptic connectivity to DSI homologues [19], [20]. Moreover, we found that the same three anatomical and neurochemical characteristics were able to uniquely distinguish a single bilaterally represented neuron in three species that do not swim with dorsal-ventral body flexions: *Melibe leonina*, *Hermissenda crassicornis*, and *Flabellina iodinea*. Because only one neuron in all of the species investigated displayed a contralateral projection into PP2 and FMRFamide and SCP_B_ immunoreactivity, we conclude that these neurons are homologous. This is the most parsimonious explanation for the same set of three characteristics identifying these neurons.

### FMRFamide and SCP_B_

Whereas we found that C2 is immunoreactive for both FMRFamide and SCP_B_, it is not clear whether C2 actually uses either peptide as a neurotransmitter. The commercially available FMRFamide antibodies are thought to stain generally for RFamides (Arg-Phe-NH_2_), but not necessarily FMRFamide specifically. The SCP_B_ monoclonal antibody is specific for antigenic sequence [50], but there is no additional molecular evidence to support the presence of native SCP_B_. Regardless of whether C2 actually uses either peptide as a neurotransmitter, the FMRFamide antisera and SCP_B_ monoclonal antibodies exhibited reliable staining patterns within species. The detection of C2 and its putative homologues via dual FMRFamide-like and SCP_B_-like immunoreactivity in conjunction with other anatomical characters helps to uniquely identify the neuron. While it remains of interest to know which peptide(s) C2 uses as a neurotransmitter, it is beyond the scope of this study.

Previous reports have documented FMRFamide and SCP_B_ immunoreactive neurons in the cerebral ganglia of opisthobranchs not discussed here. Notably, FMRFamide immunolabeling has been reported in the cerebral ganglion of the nudibranch *Phestilla sibogae* [51], and in the non-Nudipleura opisthobranchs *Bulla gouldiana* [52] and *Aplysia californica* [53]. FMRFamide gene expression in the cerebral ganglion of *Aplysia californica* has also been demonstrated via *in situ* hybridization [54]. SCP_B_ immunolabeling has been shown previously in the cerebral ganglion of *Tritonia diomedea* and *Hermissenda crassicornis* [50]. The staining patterns are similar to what we have seen in our experiments. That study also investigated two additional nudibranchs not discussed here: *Tritonia festiva* and *Dendronotus dalli* [50]. These studies reveal possible C2 homologues, but without additional information it is difficult to speculate any further on cell identity.

### The C2_Mel_ axon projection

C2*_Mel_* exhibits all of the characteristics that uniquely identify C2 in the other species tested. However, the axon projection of C2*_Mel_* showed additional branching that was not observed in the other species. It is possible that there was branching in the other species that was not captured with the tracer injections or that *Melibe* exhibits more axonal branching than the other species investigated here. Differences in axon projection patterns have been reported between other homologous gastropod neurons. One prominent example is the variability in axon morphology of the serotonergic cerebral cell, variously named the giant serotonin neuron, the metacerebral giant cell, and the giant cerebral neuron in different species; clear homologues of these neurons have been identified and axon projections have been reported in at least 12 different gastropod species [15], [16], [55]. Despite the similarities that allow these neurons to be identified as homologues, the neurons exhibit major differences in axon projections including whether the axon projects bilaterally or unilaterally. Thus, the species-differences seen in this study are not unprecedented.

### The C2 homologue in Hermissenda may be the previously identified I_b_ interneuron

Previous work in *Hermissenda* identified a bilaterally represented neuron in the anterior-lateral region of the cerebral ganglion that plays a role in ciliary crawling and foot contraction [56], [57]. This neuron, named the I_b_ interneuron (http://neuronbank.org/Her0002676) has a contralateral axon projection that closely resembles the projection pattern that we observed for C2*_Herm_* [56]. Moreover, the contralateral I_b_ interneurons are electrically coupled [56] and inhibit the serotonergic CPT neurons (http://neuronbank.org/Her0002693) [41] (homologues of the *Tritonia* DSI neurons); two characteristics that are also true of C2*_Tri_* [32], [33]. Despite the anatomical and physiological similarities between the I_b_ interneuron and C2_Herm_, we were unable to rule out the possibility that they are different neurons because we do not know if I_b_ projects through the pedal commissure or whether it is immunoreactive for FMRFamide and SCP_B_. Based on what we know, it is equally possible that the I_b_ interneuron is the white neuron just medial to C2*_Herm_*. Further experiments will be needed to determine whether the I_b_ interneuron is in fact C2*_Herm_*.

### Neuronal multifunctionality and central pattern generator evolution

While *Melibe*, *Hermissenda*, and *Flabellina* cannot perform a dorsal-ventral swim, they all possess C2. This indicates that C2 was present in a common ancestor to all Nudipleura molluscs, although we have not yet identified a C2 homologue in a species outside of this clade. It is likely that dorsal-ventral swimming arose independently several times within the Nudipleura clade because of the location on the phylogenetic tree of lineages that display this behavior (Figure 1). Therefore, the dorsal-ventral swim CPGs must have arisen from neurons (including C2) that provided another function in the ancestral brain. This function may be conserved across Nudipleura species regardless of whether they exhibit dorsal-ventral swimming.

For example, in addition to its role in the dorsal-ventral swim CPG, the C2 homologue in *Pleurobranchaea* is also involved in the suppression of feeding behaviors [19], [26]. While untested, the role of C2 in feeding behavior may be similar in other species.

A more thoroughly studied example is the role of the *Tritonia* DSIs across not just Nudipleura, but indeed more disparate opisthobranch species. These neurons are involved in feeding and locomotor or foot contraction behaviors in each of the species tested [26], [27], [37], [38], [41], [58], [59]. In *Tritonia* and *Pleurobranchaea*, the neurons are also part of the dorsal-ventral swim CPG. A separate role in crawling persists in both species, however. These examples suggest that neurons with roles in conserved behaviors can become incorporated into new circuits without losing their original function.

Now that the C2 homologues have been identified in species that cannot perform the dorsal-ventral swimming behavior, it is of interest to compare the properties of C2 and the network properties between C2 and DSI across the dorsal-ventral swimming and non-dorsal-ventral swimming species. Based on modeling and experimental studies, we know that certain network properties between C2 and DSI are crucial for producing the motor program of the dorsal-ventral swim in *Tritonia* [36], [60]. Experiments investigating those properties will be the key to understanding what differences allow these conserved neurons to reorganize and produce novel behaviors.

## Materials and Methods

### Animal collection and maintenance

*Tritonia* and *Melibe* were collected by Living Elements (Vancouver, BC, Canada). Additional *Melibe as well as Flabellina, Hermissenda and Pleurobranchaea* were collected by Monterey Abalone Company (Monterey, California, USA).

All animals were kept in re-circulating artificial seawater (Instant Ocean) tanks at Georgia State University on a 12∶12 light/dark cycle. *Pleurobranchaea* were maintained at 14°C. All other animals were kept at 10–13°C.

### Dissection

Animals were anesthetized by chilling and/or by injecting 0.33 M MgCl_2_ into the body cavity. The brain, consisting of the cerebral, pleural, and pedal ganglia, was removed from the animal and immediately pinned to the bottom of a Sylgard-lined chamber, which was superfused with saline at 4°C. Physiological saline composition was as follows (in mM): 420 NaCl, 10 KCl, 10 CaCl_2_, 50 MgCl_2_, 10 D-Glucose, and 10 HEPES, pH 7.4. For experiments where intracellular tracer injection was to be conducted, the cell bodies of the neurons were exposed by removing the connective tissue sheath from the surface of the ganglia with fine scissors and forceps. For nerve backfill experiments, the connective tissue sheath remained intact. The temperature was then raised to 14°C and 13°C for *P. californica* and *H. crassicornis*, respectively. The temperature was raised to 10–11°C for all other animals.

### Tracer injection and whole-mount immunohistochemistry

To visualize the axonal projection of the neurons, their somata were impaled with glass microelectrodes (10–50 MΩ in resistance) filled with 2–4% Neurobiotin (Vector Laboratories) and/or 2.5% biocytin (Invitrogen) dissolved in 0.75 M KCl solution. The electrodes were connected to Axoclamp 2B amplifiers (Molecular Devices). The tracer was injected via iontophoresis for 15–120 minutes (−10 to 10 nA, 1 Hz, 50% duty cycle). The injection variability was due to variability in the electrode resistance. For low resistance electrodes, high amplitude current pulses were used for short durations of time. For high resistance electrodes, smaller amplitude current pulses were applied for longer durations. After dye injection, preparations were incubated in superfusing normal saline for 12–72 hours at physiological temperatures (see above).

For biocytin nerve backfills, the cut end of the nerve was drawn into a petroleum jelly well created on top of a Sylgard block. Several drops of distilled H_2_O were added to the well and the nerve was cut again and left in H_2_O for 30 seconds. The distilled H_2_O was then replaced with a 2–2.5% solution of biocytin in 1 M KCl. The well was covered with more petroleum jelly to reduce evaporation of the dye and the preparation was incubated at 4°C for 2–48 hours. During this incubation, the dye transported retrogradely to cell bodies with axons in the nerve. After the incubation, the preparation was washed briefly in saline.

After dye injection or backfill incubations and saline washes, preparations were fixed for 12–24 hours in 4% formaldehyde in phosphate buffered saline (PBS, 50 mM Na_2_HPO_4_ in 140 mM NaCl_2_, pH 7.2). After fixation, brains were washed (20–90 minutes) with PBS and the connective tissue sheath was removed if still present. Brains were washed twice (20–30 minutes each) with 4% Triton X-100 in PBS and then incubated for 1 hour in antiserum diluent (ASD, 0.5% Triton X-100, 1% normal goat serum and 1% bovine serum albumen in PBS). This was followed by incubation in primary antiserum with Streptavidin-Alexa Fluor 594 conjugate (1∶50–1∶200, Invitrogen) added to visualize the tracer (72–120 hours): rabbit anti-FMRFamide antiserum (Immunostar) diluted 1∶1000 and/or mouse monoclonal anti-Small Cardioactive Peptide B (SCP_B_; courtesy of Stephen Kempf) diluted 1∶20 in ASD. Brains were then washed 5 times (1 hour each) with 0.5% Triton X-100 in PBS and then incubated 12–24 hours in goat anti-rabbit antiserum and/or goat anti-mouse antiserum conjugated to either Alexa Fluor 488, Alexa Fluor 594 (Invitrogen), or DyLight 405 (Jackson ImmunoResearch) diluted 1∶00 in ASD. Next, brains were washed 5 times (1 hour each) with 0.5% Triton X-100 in PBS, dehydrated in an ethanol series, cleared in methyl salicylate, and mounted on a slide with Cytoseal 60 (Richard-Allan Scientific). Brains were kept at 4°C for all the immunohistochemistry protocol before dehydration and all steps from fixation to dehydration were completed with gentle agitation.

### Imaging

Fluorescence images were obtained using confocal microscopy (LSM 510 mounted on Axiovert 100 M microscope or LSM 700 on Axio Examiner D1 microscope, Carl Zeiss, Inc.) with a 5–20× objective. Fluorophores were excited with three lasers (405, 488, and 555 nm) and fluorescent emissions were passed through a 490 nm short-pass filter to visualize DyLight 405, a band-pass filter (505–550 nm) for visualization of Alexa Fluor 488 and a 560 nm long-pass filter to visualize Alexa Fluor 594. The thickness of each confocal section was optimized and kept consistent within a preparation. Maximal projections of confocal stacks were exported as TIFF files and imported into Adobe Photoshop. Projections were assembled into a montage of the CNS and brightness and contrast were adjusted.
